# The Primary Outbreaks of Burkitt Lymphoma in the Oral Cavity. A Report of Two Cases, Review of the Literature and Dental Implications

**DOI:** 10.3390/diagnostics11122358

**Published:** 2021-12-14

**Authors:** Tomasz Kulczyk, Agata Daktera-Micker, Barbara Biedziak, Agnieszka Wziątek, Katarzyna Bednarek-Rajewska

**Affiliations:** 1 Department of Diagnostics, Poznan University of Medical Sciences, Bukowska 70, 60-812 Poznan, Poland; 2 Department of Craniofacial Anomalies, Poznan University of Medical Sciences, 60-812 Poznan, Poland; agatamicker@icloud.com (A.D.-M.); biedziak@ump.edu.pl (B.B.); 3 Department of Pediatric Oncology and Transplantology, Poznan University of Medical Sciences, 60-812 Poznan, Poland; agawziatek@gmail.com; 4 Department of Clinical Pathology, Poznan University of Medical Sciences, 60-812 Poznan, Poland; krajewska@ump.edu.pl

**Keywords:** Burkitt’s lymphoma, dental abscess, oral cavity, paediatric, starry sky pattern

## Abstract

Two cases of Sporadic Burkitt’s lymphoma in children aged 11 and 8 years with primary symptoms in the oral cavity are reported. The first symptoms of the disease appeared in the oral cavity and were initially misdiagnosed as an inflammatory condition in one case and incidental findings not associated with the primary reason for visiting the dentist’s office in the second case. Biopsies of the lesions revealed the actual cause of the observed changes and contributed to the prompt initiation of polychemotherapy treatment. A review of current literature presents the known symptoms of Burkitt’s Lymphoma in the oral cavity and the available diagnostic methods. The role of the primary care physicians—the pedodontist and paediatrician—in the diagnostic and therapeutic process is discussed.

## 1. Introduction

Sporadic Burkitt’s lymphoma, also called American or idiopathic lymphoma, is a malignant tumour of B-cell lymphocytes classified as a subgroup of non-Hodgkin’s lymphoma. The African or endemic and HIV-associated types are the other two clinical variants of the disease. Initially, the disease was delineated in the early 20th century by Albert Cook in eastern Africa, and later, in 1958, it was presented and published by Denis Burkitt. He described it as a sarcoma involving the jaws [1]. Sporadic Burkitt’s lymphoma (SBL) is not related to geographical region, accounts for about 30 to 40% of lymphomas in children [2,3] in well-developed countries, and occurs at a frequency of 1–3 cases per million [4]. The average onset time for SBL is 12 years, with the male-to-female ratio between 2:1 to 4:1 [5,6]. The most common location of SBL is the abdomen, Waldeyer’s ring, ovaries, and kidneys. The maxillo-facial location of SBL can be seen in about 12 to 30% of cases. In this paper, we present two cases of Burkitt’s lymphoma in boys aged 11 and 8 years with primary symptoms in the oral cavity.

## 2. Clinical Cases

**Figure 1:**
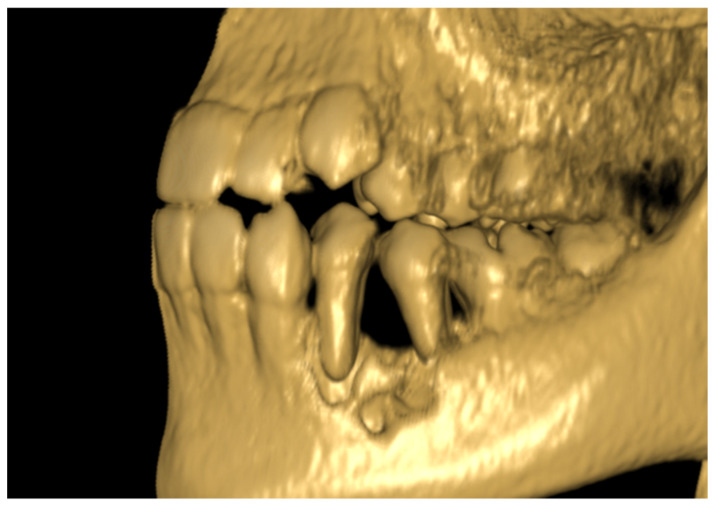
Patient, 11 y.o.: 3D volumetric reconstruction from CBCT examination: vertical bone loss and cortical bone destruction in the region of teeth 34 and 35.

**Figure 2:**
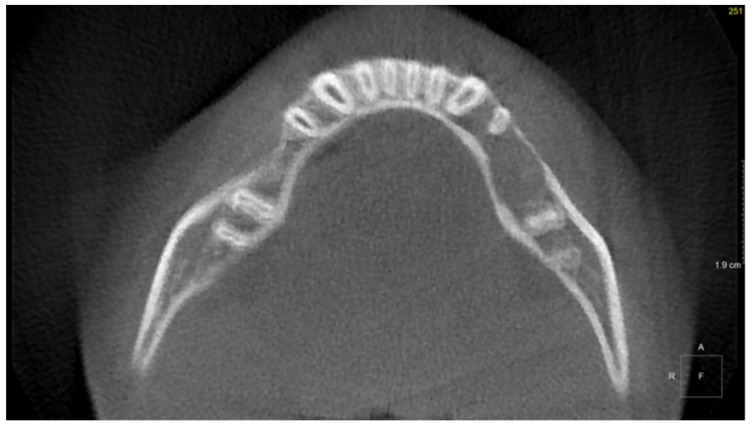
Patient, 11 y.o.: Axial image from CBCT examination with loss of trabecular pattern and thinning of cortical lingual and buccal plates in the region of teeth 33, 34, and 35.

**Figure 3:**
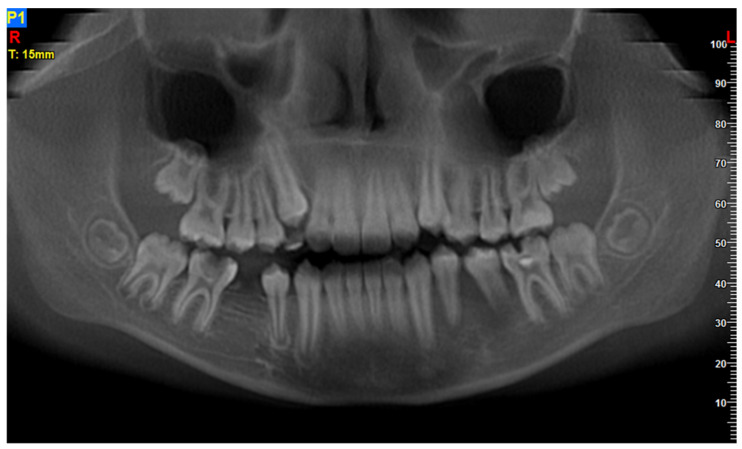
Patient, 11 y.o.: Panoramic reconstruction from CBCT examination. Rarefication of cancellous bone in the apical region of tooth 35 with apical resorption and the appearance of “floating in the air”.

The 11-year-old patient was referred to the Children’s Dental Clinic with suspected inflammation of the periapical tissues of the region of the left premolar of the mandible. Clinical evaluation revealed hypermobility of teeth 34 and 35 (FDI) with enlargement and redness of gingival tissue of both the vestibular and lingual side of the alveolus with pain symptoms. Teeth with no signs of caries lesions were not tender to percussion, the vitality tests were inconclusive, and no palpable lymphadenopathy was noticed. A cone beam computed tomography (CBCT) examination (NewTome 3G, QR Verona, Italy, 110 kV, 2 mA, 18 s) revealed root resorption of tooth 35 and an area of extensive demineralisation in the region of 33–36 with loss of trabecular bone pattern, destruction of buccal, and thinning of the lingual cortical plates (Figure 1, Figure 2 and Figure 3). On the basis of the clinical examination and the unusual features of the X-ray examination, the diagnosis was extended to include a biopsy of soft and hard tissue. A histological examination revealed Burkitt’s lymphoma. An extent examination using 2-deoxy-2-[fluorine-18] fluoro- D-glucose integrated with computed tomography (18F-FDG PET CT), magnetic resonance imaging (MRI), and ultrasonography (USG) revealed a disseminated tumour process with infiltrates in the left palatine tonsil, head of the pancreas, stomach wall, and right pleura. On the basis of the results of the extended examination and according to the European Intergroup for Childhood Non-Hodgkin Lymphoma (EICNHL-COG Inter-B-NHL-2010) therapeutic protocol, the patient was classified into the B-High therapeutic group, and a 7-day cytoreductive prephase COP (Encorton, Cyclophosphamide, and Vincristine) was initiated. After completing the prephase, significant regression of the primary lesion was observed. Subsequently, the patient completed two cycles of R-COPADM (Rituximab, Vincristine, Methotrexate 3 g/m^2^, Cyclophosamide, Doxorubicin, and Encorton) and two cycles of R-CYM (Rituximab, Encorton, Methotrexate, and Cytarabine) treatments. The patient responded well to treatment, and remission was achieved. Now, five years after initial diagnosis, he is undergoing regular check-ups, including dental examinations. The intraoral lesion resolved completely because of chemotherapy, and no surgical treatment was performed (except for initial biopsy taking).

**Figure 4:**
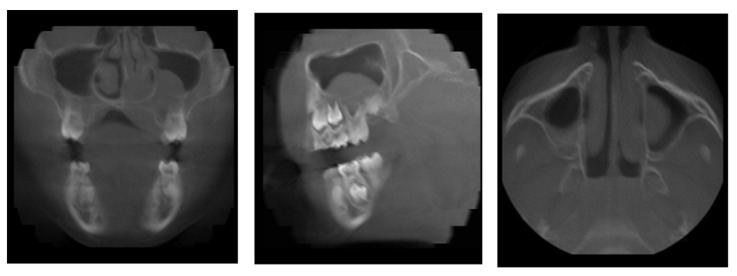
Patient, 8 y.o.: Coronal, sagittal, and axial reconstruction from CBCT examination. Soft-tissue mass covering the area of the hard palate and extending into a maxillary sinus and a nasal cavity.

**Figure 5:**
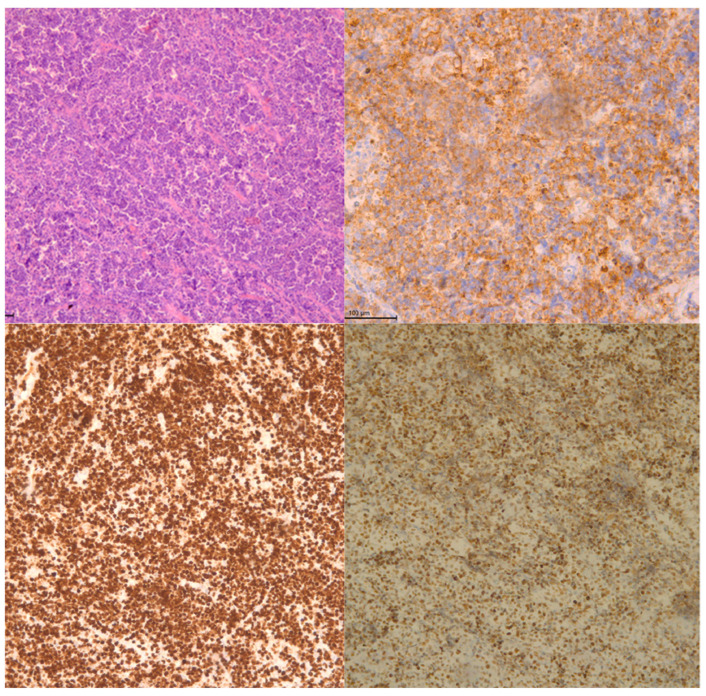
Patient, 8 y.o. Microscopic evaluation of specimen: Burkitt’s lymphoma infiltrating the mandible: A. Haematoxylin and Eosin, magnification ×100. B. Staining for CD10, magnification ×200. C. Proliferation rate near 100% by KI-67, magnification ×100. D. BCL6 expression in neoplastic cells, magnification ×100.

**Figure 6:**
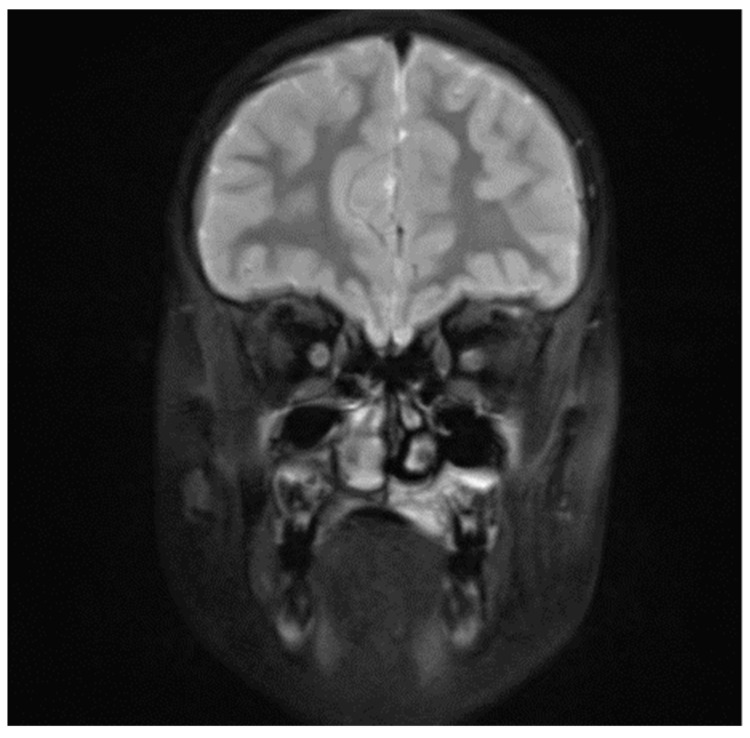
Patient, 8 y.o.: Coronal section MRI T2 fast spin fat-saturation image, increase in signal intensity with thickening of the palatal mucosa on the left side.

The 8-year-old patient was referred to the Children’s Dental Clinic because of trauma of front upper central incisors. Hypermobility of the left upper incisor with swelling and bloody petechiae of the upper lip were noticed. Because the periapical X-ray was inconclusive, a CBCT examination (NewTome 3G, 110 kV, 2 mA, 18 s) of the maxilla was performed to evaluate the possible presence of a fracture of either the root or the alveolar bone. CBCT ruled out the presence of a fracture in the front upper region but revealed the presence of a soft-tissue mass covering the area of the hard palate and extending into a maxillary sinus and a nasal cavity, which had dimensions of 25 × 25 × 20 mm (Figure 4). Clinical re-examination showed a dome-shaped enlargement of the palate extending from the midline to the palatal aspect of tooth 26 (FDI). A non-shifting mass of medium consistency was covered with pale, normal-looking soft tissue. The adjacent teeth showed normal vitality and mobility, and no palpable lymphadenopathy was observed. The patient underwent an open biopsy of the palatal mass. Histopathological examination revealed a high-grade B-cell lymphoma (Figure 5). The patient then underwent an extent-of-disease evaluation. MRI examination in the TSE, T1 and T2, and fluid-attenuated inversion recovery (FLAIR DWI) sequences was performed. An increase in signal intensity with thickening of the palatal mucosa on the left side was observed with a blurred pattern of the alveolar bone in the region of teeth 26 and 27 and post-contrast enhancement (Figure 6). A positron emission tomography (PET-CT) examination did not reveal the presence of other outbreaks of the disease in the regions of the head and neck, abdominal cavity, pelvis, or skeleton. On the basis of the results of the extended examination, the patient was classified into stage III and the low/intermediate treatment group according to the EICNHL-COG Inter-B-NHL-2010 protocol, and a 7-day cytoreductive prephase COP was initiated. Subsequently, the patient completed two cycles of COPADM and two CYM cycles without Rituximab. Because of an unsatisfactory response to the chemotherapy, supplementary treatment was introduced with two additional cycles of R-CYVE (Rituximab, Cytarabine, and Etoposide). Finally, the patient responded well to the therapy, and remission was achieved. Currently, seven years after the initial diagnosis, he undergoes regular check-ups, including dental examinations. The intraoral lesion resolved completely because of chemotherapy, and no surgical treatment was performed.

## 3. Discussion

Sporadic Burkitt’s lymphoma is a poorly differentiated lymphocytic lymphoma that appears worldwide without a geographic or climate predilection [7]. It affects mainly older children (with an average onset age of 12 to 14 years), occurring at a frequency of 1–3 cases per million [5,6]. In SBL, the most common symptom of the disease is the presence of an abdominal mass, while the jaws and sinuses are less frequently involved (12 to 30% of cases) and mostly seen as isolated lesions [8]. By comparison, in cases of endemic BL (EBL), which mainly affects Central Africa, a higher incidence (10 per million) and an earlier onset of symptoms (7 years of age) is observed [4]. In addition, in EBL cases, lesions in the jaws, particularly maxillary lesions, are reported in up to 75% of cases, with frequent presence in several quadrants [5,9,10]. An SBL lesion in the jaw region usually presents clinically as an extranodal tumour [3,11] composed of a monoclonal proliferation of undifferentiated B cells. In the case of tumours located in the oral cavity, clinical symptoms may involve increased tooth mobility, tooth loss [6], swelling of the mucosa resembling the formation of a periapical abscess [12], or swelling of the mucosa with a firm, non-fluctuant mass [13]. In several reports, a regional hard swelling was seen with or without pain symptoms [13,14,15]. When the tumour was located in the mandible, hypoesthesia of the right lower lip was also occasionally noticed. Two reports indicated lip anaesthesia and chin numbness as the sole presenting symptoms of SPL [16,17]. For cases located in the maxilla, an enlarging mass sometimes led to orbital swelling, proptosis, diplopia, and pruritus [10,13]. Radiographic findings of SBL in the craniofacial region may vary depending on the technique employed and the region of interest. Commonly used 2D X-ray modalities, such as periapical and panoramic X-rays, may reveal areas of diffused radiolucency without a regular trabeculae pattern, while an occlusal X-ray is used to confirm hard tissue swelling [17]. A radiological appearance of teeth as “floating in the air” was also noticed [4,12]. However, it has been reported [9] that panoramic images may be of limited usefulness when the lesion is in the lateral region of maxillary alveolar bone. Superimposition of anatomical structures in this region may hide the presence of the lesion despite its clear clinical presence. Three-dimensional modalities used for the diagnosis of SBL in the facial region include CBCT, CT, and MRI. CBCT examination in the mandible and maxilla can reveal osteolytic lesions with poorly defined margins and a mottled, permeative pattern of bone destruction [18]. Sometimes, balloon-like bone expansion is present with thinning or destruction of the cortical plates and a spiculated periosteal reaction. CT findings of lesions in SBL show soft-tissue destructive mass with erosion of the surrounding bone and invasion into neighbouring spaces with a mild to moderate enhancement of the associated soft tissue mass after contrast administration [18]. MRI examinations are usually performed in protocols of contrast MRI of the face with fat saturation, including short tau inversion recovery (STIR) [18,19]. T1W1 images show intermediate to low signals, and T1 post-Gd shows contrast enhancement with a good definition of the soft tissue component. In every case of SBL, it was noticed that, in the oral cavity region, an extended examination using 18F-FDG PET CT and MRI was be performed to disseminate the tumour process in other regions, including the abdomen, Waldeyer’s ring, ovaries, and kidneys [3].

A variety of signs and symptoms of SBL in the craniofacial region should be underlined when the dentist’s and paediatrician’s roles in the diagnosis of SBL are considered. Some more commonly seen conditions for this group age, such as acute periapical abscess, periodontal inflammation, and the presence of foreign bodies in the oral cavity, are usually considered the initial diagnosis when some enlarging of the soft or hard tissue mass is noticed. In such circumstances, the clinical and radiological examination should confirm the initial diagnosis. During examination, attention should be paid to the absence or contradiction of the typically associated disease features. Signs of a periapical inflammation-like condition in the absence of a caries lesion on a tooth is a good example. Another is the mobility of the tooth in the absence of periodontitis. Atypical tooth morphology with shorter-than-expected and widened roots should arouse interest and provoke a search for the causes of this state. The doctor conducting the examination should also be alert to the sudden onset of symptoms, such as regional paresthesia, and be aware that regional lymphadenopathy is only observed in approximately 25% of cases of SBL [20]. When the main clinical issue is local tissue enlargement, the differential diagnosis for SBL should also include some rare conditions, such as eosinophilic granuloma, leukaemia, osteomyelitis, and rhabdomyosarcoma. Moreover, in cases with teeth mobility, the list of possible diagnoses includes generalised juvenile periodontitis histiocytosis, Papillon-Lefèvre syndrome, hypophosphatasia, acrodynia, cyclic neutropenia, and vitamin-D resistant rickets [7].

## 4. Conclusions

The dentist’s role is primarily to be vigilant in situations that do not correspond to the typical course of disease in the oral cavity. In case of suspicion, the dentist should act quickly, which means, above all, immediate referral of the patient for further diagnostic tests performed in specialised medical institutions. In most cases, the lesion in the oral cavity does not require dental treatment and resolves in the course of combined treatment with polychemotherapy. Because of very high replicative activity, SBL is particularly sensitive to some polychemotherapy schemes. The prognosis of SBL in children depends on the extent of the disease, age, and time of diagnosis. In children, the prognosis is very good, with a survival rate of 90–100% for the early stages [6,9,18].

## Data Availability

Data available on request.
